# Simplifying Pharmacokinetics, Applying it to Drug and Dosage Form Development, and Making Drug Dosage Decisions in Clinical Medicine: The Adaptation of Kirchhoff’s Laws from Physics

**DOI:** 10.1208/s12248-025-01099-6

**Published:** 2025-07-03

**Authors:** Leslie Z. Benet, Jasleen K. Sodhi

**Affiliations:** 1 Department of Bioengineering and Therapeutic Sciences, Schools of Pharmacy and Medicine, University of California San Francisco, San Francisco, California 94143-0912, USA; 2 Present Address: Department of Drug Metabolism and Pharmacokinetics, Septerna, South San Francisco, California 94080, USA

**Keywords:** bioavailability, clearance, drug delivery, Kirchhoff’s Laws, pharmacokinetics

## Abstract

Over the past three years, we have published a series of nine manuscripts demonstrating that all relevant pharmacokinetic relationships may be simply derived independent of differential equations, offering an alternative to traditional pharmacokinetic analyses. These derivations are based on an understanding of parallel and in series rate-defining processes, and account for all relevant drivers, including organ blood flow and drug delivery clearance kinetics, across both linear and nonlinear scenarios. In this tutorial, we present the simple derivation of renal clearance and hepatic clearance directly relevant to clinical pharmacokinetics, as applied to making drug dosing decisions based on measures of systemic exposure. We further advocate for a more streamlined and practical approach to teaching and applying clinical pharmacokinetics, noting that compartmental modeling, protein binding in hypothetical compartments, trapezoidal AUC calculations, and alternative volume of distribution parameters, aside from (the unfortunately misnamed) volume of distribution steady-state, often overcomplicate pharmacokinetics in practice. The key advantage of this simplified methodology is the ability to directly incorporate clearance from the drug delivery site into systemic pharmacokinetic relationships. This enables a clear understanding of how entering clearance can influence systemic AUC, helping explain: enhanced pharmacodynamic outcomes of slow drug delivery *versus* immediate-release formulations; systemic bioavailability measures exceeding unity, statistically significant discrepancies between urinary and systemic bioavailability measures; and changes in renal clearance as a function of drug clearance from the delivery site. These key concepts are illustrated by applying the proposed methodology to an example drug, analyzing all relevant clinical pharmacokinetic relationships required for dosing decisions.

## Introduction

With the development of analytical methods capable of accurate and sensitive measurement of *in vivo* drug levels in humans and animals, pharmacokinetic principles and analyses were subsequently introduced. Founded on principles utilized in chemistry, differential equations, derived based upon mass balance relationships, were employed to describe the time-course of drug concentrations, and the rate constants governing that time-course. However, these early approaches provided little information that could be leveraged in clinical pharmacology to make drug dosage decisions for a new molecular entity (NME), or for adjustments of dosage in an individual patient due to disease states, drug-drug interactions, pharmacogenomics or physiologic variation. Rather, dosing decisions for the overwhelming majority of drugs are based on exposure, measured as area under the systemic concentration–time curve (AUC). Exposure is a function of two pharmacokinetic parameters, clearance (CL), a measure of the body’s ability to eliminate drug with units of volume per unit time, and bioavailability (F), a measure of the extent of an administered dose that reaches the systemic circulation intact as given (1) in Eq. 1. For a single dose of the drug, where concentrations are measured over all time (AUC_0→∞_), or for multiple doses of a drug following first-order kinetics at steady state, then:

(1) $$AUC_{single\;dose\;0\to \infty \;or\;during\;a\;dosing\;interval\;at\;steady-state}=\frac{F•Dose}{CL}$$

Therefore, to predict exposure to enable drug dosing decisions in clinical medicine, one must know the value of CL determined following an iv bolus dose of drug where complete bioavailability is assumed (i.e., F = 1) or CL/F for alternate routes of administration.

In this tutorial, we aim to: (i) identify the key gaps in existing pharmacokinetic analysis approaches, (ii) present a simple derivation of renal and hepatic clearance, directly relevant to clinical pharmacokinetics, using principles based on application of Kirchhoff’s Laws from Physics, (iii) illustrate the advantages of the proposed methodology through application to an example drug, and (iv) advocate for a more streamlined and practical approach to the teaching and application of clinical pharmacokinetics.

## The Components of Clearance

For the vast majority of drugs, CL, the measure of the body’s ability to eliminate drugs from the systemic circulation, occurs primarily in the liver (via metabolism or excretion of unchanged drug into the bile) and/or in the kidney (via elimination into the urine). Renal elimination via urine can predominantly occur by two processes: a) excretion of drug by glomerular filtration, calculated as the fraction of drug unbound in blood (f_uB_) multiplied by the glomerular filtration rate (GFR), where GFR is approximately 120 ml/min in a healthy young 70 kg man (~ 108 ml/min in a healthy young 70 kg woman), but which decreases with age as kidney tubules lose activity. And by b) active secretion of drug into urine via transporters minus passive and active reabsorption of drug from urine back to the systemic circulation. There are other possible mechanisms for drug elimination, for example, into the exhaled breath for low vapor pressure drugs (which can be measured in patients) and a number of potential processes that cannot or are very difficult to measure in patients (e.g., intestinal secretion of drug from the circulation directly into the intestine with an unmeasurable degree of reabsorption, extra-hepatic metabolism by organs other than the liver, excretion in the sweat, elimination dependent on urine flow rate). However, these are less-common mechanisms of clearance that go beyond the general approach presented here.

## The Application of Kirchhoff’s Laws to Simply Derive Clearance Relationships

In 2022, we recognized that Kirchhoff’s Laws, commonly taught in all introductory physics courses, could be adapted to simplify the derivation of clearance and total rate constants of elimination in pharmacokinetics, independent of the differential equations approach employed for the past century (2). Here as applied to clearance, when two or more rate-defining elimination processes operate in parallel, with each clearance value having no effect on the other measured clearance values, the total clearance is equal to the sum of the individual parallel rate-defining processes, as given in Eq. 2.


(2) $$CL_{total}=CL_{rate-defining\;parallel\;process\;1}+CL_{rate-defining\;parallel\;process\;2}+\ldots$$

For example, in the liver there are two elimination processes, metabolism and biliary excretion. The metabolic clearance value has no effect on the biliary clearance value, and vice versa. Therefore, they are parallel processes. Note that in pharmacokinetics, the positional characteristics of each process have no relevance to the definition of “parallel”, as the biliary process is proximal to the metabolic process. In the kidney, there are also two elimination processes, glomerular filtration and the difference between tubular secretion clearance and tubular reabsorption clearance. The value of glomerular filtration clearance has no effect on the value of the tubular clearance processes, and vice versa. Therefore, these are also parallel processes, although tubular elimination processes (secretion/reabsorption) occur proximal to glomerular filtration.

However, when two or more rate-defining processes operate in series, meaning that each clearance value in the series may be influenced by the value of the preceding clearance term, then the total clearance is determined by the sequential effect of these processes. That is, the inverse of the total clearance is equal to the sum of the inverse of each in series rate-defining processes, as given in Eq. 3.


(3) $$\frac{1}{CL_{total}}=\frac{1}{CL_{rate-defining\;in\;series\;process\;1}}+\frac{1}{CL_{rate-defining\;in\;series\;process\;2}}+\ldots$$

In adapting Kirchhoff’s Laws to *in vivo* pharmacokinetic relationships, it is critical to understand the concept of a rate-defining process. This limitation is not present when applying Kirchhoff’s Laws to physical systems, as only rate-defining processes are considered by default. However, *in vivo* drug disposition often involves many additional processes that may not be considered rate-defining (e.g., distribution into hypothetical peripheral compartments). Adapted from our recent publication (3): A rate-defining process is defined by a parameter that describes an elimination or movement process for which it is possible under certain conditions that the total clearance may be equal to this parameter. For example, a rate-defining clearance process for hepatic elimination could be hepatic blood flow, i.e., the rate at which the drug arrives to the liver is the maximum value that hepatic elimination can be. Thus, for a very high hepatic clearance (CL_H_) drug, the total CL_H_ could equal the rate-defining process hepatic blood flow (Q_H_). Furthermore, for a series of chemical reactions in a beaker, the elimination clearance for the parent drug could be the minimum value rate-defining process for all subsequent metabolic steps. That is, none of the clearance measurements for subsequent metabolites can be greater than that for the parent drug. The critical aspect of our approach is that only rate-defining processes can be combined to correctly determine the overall clearance following Kirchhoff’s Laws. Passive permeability by itself, no matter how slow, cannot be a rate-defining process for elimination; clearance will never be equal to passive permeability. This is due to the mechanistic definition of passive permeability, as passive permeability clearance parameters are bidirectional and equal, and therefore, on its own, it cannot contribute to net directional flux or establish a net directional clearance. Undoubtedly, when active membrane transport is a rate-defining process, passive permeability can influence the magnitude of directional flux and contribute to overall influx or efflux directional clearances. However, in the context of net flux across a membrane, its contributions cancel out. As a result, it does not affect the overall clearance and therefore cannot independently define the rate of elimination. Thus, following iv bolus dosing for a drug exhibiting multiple exponential terms due to distribution into unmeasurable peripheral compartments, clearance is never a function of the passive permeability rate constants or clearances between these hypothetical spaces and the measurable systemic concentration. As subsequently addressed, when hepatic basolateral transporters influence permeability and active influx exceeds active efflux, this can be a rate-defining process. However, this does not apply when active efflux exceeds active influx. That is, clearance can never be defined singly as active efflux minus the smaller active influx, as this would result in negative value, which is not physiologically meaningful. Note that inherent in this definition is the requirement that, under some condition, a rate-defining step can be experimentally measured. In other words, when deriving *in vivo* clearance, each rate-defining step can potentially be measured *in vivo* for a given drug.

## The Simple Universal Relationship to Derive Clearance Equations

A very simple general approach (4) can be utilized to derive all clinically relevant clearance relationships as given in Eq. 4.


(4) $$\frac{1}{CL_{total}}=\frac{1}{CL_{entering}}+\frac{1}{CL_{leaving}}$$

Now we utilize Eq. 4 to characterize the clinically relevant characteristics for hepatic and renal clearance, as well as for clearance measures when drugs are administered via routes other than iv bolus dosing.

## Renal Clearance

In deriving renal clearance (CL_R_) following an iv bolus dose, CL_leaving_ is the sum of two parallel processes, glomerular filtration (CL_filtration_) and the difference between renal transporter-related secretion (CL_R,secretion_) and renal reabsorption (CL_R,reabsorption_), while CL_entering_ is kidney blood flow (Q_R_), as given in Eq. 5

(5) $$\frac{1}{CL_{R}}=\frac{1}{Q_{R}}+\frac{1}{CL_{filtration}+(CL_{R,secretion}-CL_{R,reabsorption})}$$

Prior to our introduction of this relationship, Eq. 5 had never been previously presented since it cannot be derived using differential equations to incorporate renal blood flow. It could be argued that for most renally excreted drugs, renal blood flow will have minimal impact and need not be considered. However, this is not always true. For example, the measured CL_R_ of metformin is approximately 600 ml/min in healthy young humans and with negligible plasma protein binding (f_uB_ ≈ 1.0), CL_filtration_ = GFR ≈ 120 ml/min. Therefore, previously the secretory clearance of metformin $\left(CL_{R,secretion}-CL_{R,reabsorption}\right)$ was assumed to equal approximately 480 ml/min. However, when kidney blood flow is included (Q_R_ ≈ 1200 ml/min) as in Eq. 5, the secretory clearance is instead found to be 1080 ml/min, a value greater than twofold higher than estimated with the previous approach to estimating renal clearance. Thus, we have been significantly underestimating the importance of tubular transport for drugs exhibiting moderate renal clearance values, such as metformin. This has significant implications when using *in vitro* measurements to predict *in vivo* parameters, i.e., IVIVE (*in vitro* – *in vivo* extrapolation). It is also highly relevant in predicting and defining the extent of transporter drug-drug interactions.

## Hepatic Clearance

In deriving hepatic clearance (CL_H_) following an iv bolus dose, CL_leaving_ is the sum of two parallel processes, metabolism (CL_met_) and biliary excretion (CL_bil_), while CL_entering_ is liver blood flow (Q_H_) and the difference between hepatobiliary membrane influx (CL_H,influx_) and hepatobiliary membrane efflux (CL_H,efflux_), two in series entering processes, as given in Eq. 6

(6) $$\frac{1}{CL_{H}}=\frac{1}{Q_{H}}+\frac{1}{\left(CL_{H,influx}-CL_{H,efflux}\right)}+\frac{1}{CL_{met}+CL_{bil}}$$

For hepatic clearance, the effects of protein binding must also be considered by inserting fraction unbound in blood (f_u,B_) and intrinsic (int) clearances thereby converting Eq. 6 to

(7) $$\frac{1}{CL_{H}}=\frac{1}{Q_{H}}+\frac{1}{f_{u,B}•(CL_{int,H,influx}-CL_{int,H,efflux})}+\frac{1}{f_{u,B}•CL_{H,int}}$$

where CL_H.int_ is the sum of the intrinsic metabolic and biliary clearances. Above, we have simply derived the renal and hepatic clearance equations based on parallel and in series rate-defining processes independent of differential equations. The justification for these derivations has been detailed in our series of 9 published papers, including critical analysis of published clinical data, culminating in our 2025 Pharmacological Reviews manuscript (5).

## The Application of Kirchhoff’s Laws to Derive Rate Constant Relationships

Just as this methodology of parallel and in series rate-defining processes can be applied to derive total clearance equations, it can also be applied to the derivation of total rate constants of elimination, as described in Eqs. 8-10.


(8) $$k_{total}=k_{rate-defining\;parallel\;process\;1}+k_{rate-defining\;parallel\;process\;2}+\ldots$$

(9) $$\frac{1}{k_{\mathrm{total}}}=\frac{1}{k_{\mathrm{rate}-\mathrm{defining}\;\mathrm{in}\;\mathrm{series}\;\mathrm{process}\;1}}+\frac{1}{k_{\mathrm{rate}-\mathrm{defining}\;\mathrm{in}\;\mathrm{series}\;\mathrm{process}\;2}}+\ldots$$

(10) $$\frac{1}{k_{total}}=\frac{1}{k_{rate-defining\;entering\;process}}+\frac{1}{k_{rate-defining\;leaving\;precess}}$$

Equations 9 and 10 are mathematically equivalent, with Eq. 9 illustrating the general case of in-series processes and Eq. 10 offering a more intuitive framing based on process directionality.

## Drug Input and Mean Residence Time Concepts

Until now, we have only considered iv bolus dosing, but the great majority of clinical pharmacokinetic data are following oral (po) or other drug dosing routes, e.g., intramuscular (IM), subcutaneous (SubQ). Mean residence time (MRT, i.e., the average time a drug is measurable in the systemic circulation) concepts allow simple analysis of such data (6) by including mean absorption time (MAT).


(11) $$MRT_{system\;oral\;dose}=MRT_{iv\;bolus}+MAT$$

In fact, for first order systems, MRT values are actually the inverse of the single first order rate constants describing the process.


(12) $$\frac{1}{k_{system\;oral\;dose}}=\frac{1}{k_{system\;iv\;bolus}}+\frac{1}{k_{absorption}}$$

As shown, Eq. 12 follows the same format as Eqs. 9 and 10. Thus, the field has been using an approach analogous to the in series derivations in pharmacokinetics since Yamaoka *et al.* (6) introduced mean residence time concepts in 1979. Then solving Eq. 12 for k_system oral dose_ gives

(13) $$k_{system\;oral\;dose}=\frac{k_{system\;iv\;bolus}•k_{absorption}}{k_{system\;iv\;bolus}+k_{absorption}}=\frac{k_{system\;iv\;bolus}}{1+\frac{k_{system\;iv\;bolus}}{k_{absorption}}}$$

Only if k_absorption_ is significantly greater than k_system iv bolus_ will the pharmacokinetic terminal concentration time-curve have the slope of k_system iv bolus_. In many cases this will be true in particular for drugs with fast absorption, but frequently the absorption rate constant can be very slow, often deliberately for controlled release dosage forms. Hence, Eq. 13 mathematically explains the flip-flop model. If k_absorption_ is not much greater than k_system iv bolus_, the terminal slope (k_system iv bolus_) is given by Eq. 13. And if k_absorption_ is much slower than k_system iv bolus_, then the terminal concentration time curve will reflect the absorption rate constant.

## Clearance from the Absorption Site (CL_absorption site_) and Absorption Site Volume of Distribution (V_absorption site_)

However, drug dosing decisions are made based on clearance values, not rate constants. The clearance equivalent of Eq. 12 is

(14) $$\frac{1}{{\mathrm{CL}}_{\mathrm{system}\;\mathrm{oral}\;\mathrm{dose}}}=\frac{1}{{\mathrm{CL}}_{\mathrm{system}\;\mathrm{iv}\;\mathrm{bolus}}}+\frac{1}{{\mathrm{CL}}_{\mathrm{absorption}\;\mathrm{site}}}$$

Clearance from the absorption site is a parameter that had not been considered prior to Wakuda *et al.* (7). For first order absorption, it is simply the product of k_absorption_ and the volume of distribution of drug in the absorption site (V_absorption site_), with the latter being an additional pharmacokinetic parameter that we introduced (7). V_absorption site_ is the non-physiological parameter that defines the amount of drug at the absorption site divided by the concentration of drug at the absorption site. V_absorption site_ has no more physiologic relevance than the volume of distribution steady state (V_ss_), but will certainly not be equal to V_ss_, as we previously described (4, 7). Then solving for CL_system oral dose_

(15) $$CL_{system\;oral\;dose}=\frac{CL_{system\;iv\;bolus}•CL_{absorption\;site}}{CL_{system\;iv\;bolus}+CL_{absorption\;site}}=\frac{CL_{system\;iv\;bolus}}{1+\frac{CL_{system\;iv\;bolus}}{CL_{absorption\;site}}}$$

The clearance measured following oral dosing will only equal CL_system iv bolus_ if CL_absorption site_ is significantly greater than CL_system iv bolus_. In most cases this will be true, but frequently the clearance from the absorption site can be very slow, often deliberately for controlled release dosage forms. We will return to the implications of Eq. 15 after discussing bioavailability.

## Bioavailability, Oral Absorption, First Pass Intestinal and First Pass Hepatic Metabolism

**Fig. 1:**
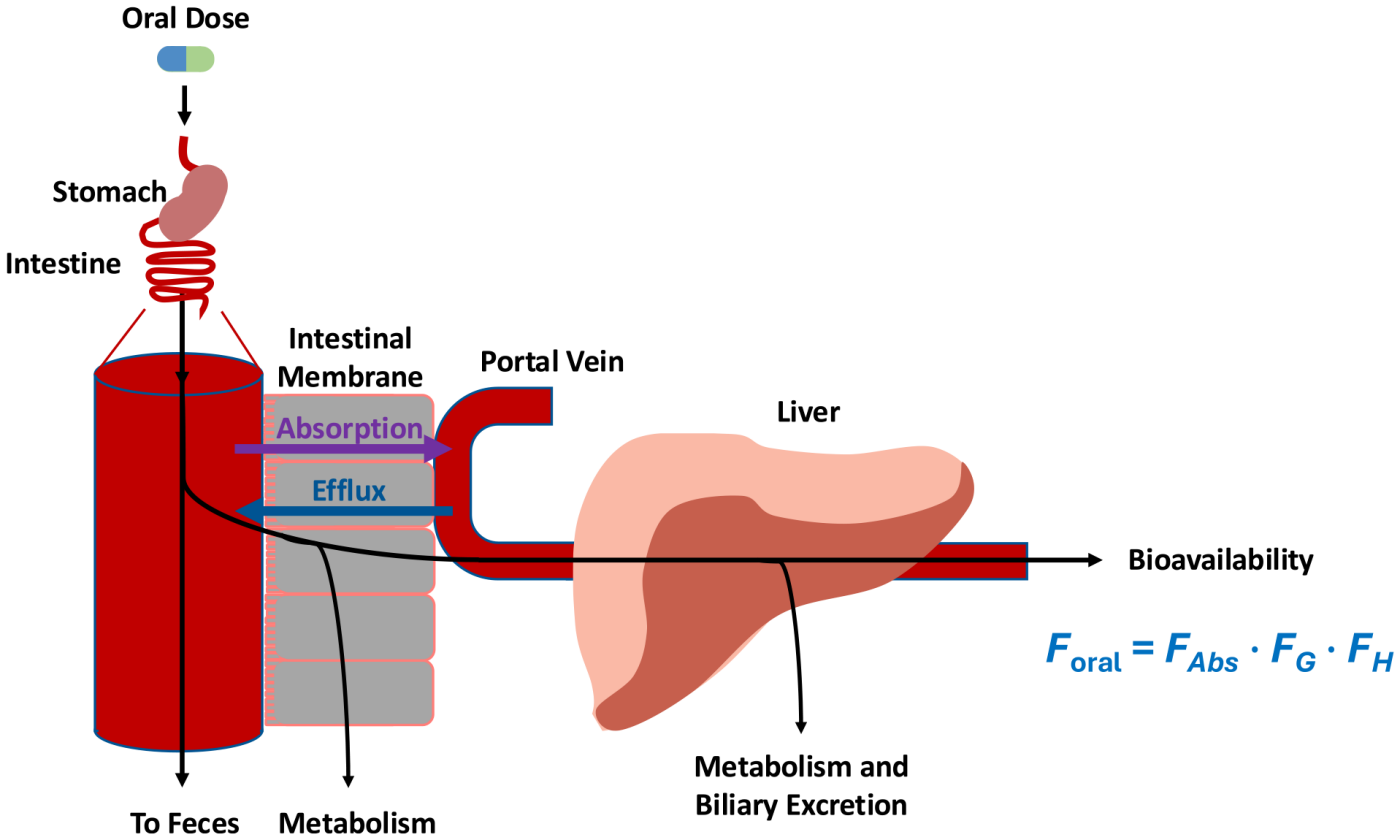
Schematic depiction of oral dosing processes leading to measures of bioavailability. Drug is absorbed from the intestinal lumen usually by passive processes for high permeability substrates and via passive plus active transport for poor permeability compounds and potentially effluxed back into the lumen. Fraction of the dose absorbed (F_Abs_) is a measure of total drug absorbed that is not effluxed back into the intestinal lumen. The first-pass fraction of the dose passing intact though the gastrointestinal lumen (F_G_) is a function of intestinal metabolism, primarily due to CYP3A and UGTs. The hepatic bioavailability (F_H_) is a measure of the portion of the dose that is not metabolized or excreted unchanged in the bile on the first pass of drug through the liver

Bioavailability of an oral dose (F_oral_), the fraction of an oral dose that reaches the systemic circulation intact, is the product of the fraction of the dose absorbed (F_Abs_), the fraction of the dose that passes intact through the gut (F_G_) and the liver (F_H_), as depicted schematically in Fig. 1, and given in Eq. 16

(16) $$F_{oral}=F_{Abs}•F_{G}•F_{H}$$

As most drugs are lipophilic, they readily pass from the aqueous intestinal fluids into the intestinal membrane as reflected by the purple absorption arrow. However, the body has protective mechanisms against xenobiotics entering the systemic circulation. In the intestine, primarily two efflux transporters, P-glycoprotein (P-gp) and Breast Cancer Resistance Protein (BCRP), pump xenobiotics that have entered the enterocyte back into the intestinal lumen, as reflected by the blue efflux arrow. Thus, F_Abs_ is the resultant fraction of the dose absorbed that is not effluxed back into the lumen. There are also metabolic enzymes expressed in the intestine (predominantly CYP3A and UGTs) that decrease bioavailability via metabolism, as reflected in F_G_. And finally, all orally administered drug must pass through the liver before reaching the systemic circulation, where the drug may be metabolized by multiple enzymes as well as be excreted in the bile, as reflected in F_H_. Now returning to Eq. 1, rewritten as Eq. 17

(17) $$AUC_{single\;dose\;0\to \infty \;or\;during\;a\;dosing\;interval\;at\;steady-state}=\frac{F•Dose}{CL}$$

Following an iv bolus dose, where F = 1, one can measure AUC and then determine the value of CL based on the dose administered.

Following an oral dose, F can be calculated from Eq. 17 by assuming that CL between iv bolus and oral doses is unchanged, and by making any adjustment for differences in iv *vs* po doses. Once knowing total clearance and measuring renal clearance (the amount of drug eliminated unchanged in the urine divided by the systemic exposure, AUC) one can also determine CL_H_ since

(18) $$CL_{total}=CL_{R}+CL_{H}$$

Then, it is possible to estimate F_H_, since the liver extraction ratio (ExR_H_) is given as the ratio of hepatic blood clearance to hepatic blood flow.

(19) $$ExR_{H}=\frac{CL_{H}}{Q_{H}}$$

and F_H_ is defined as 1-ExR_H_, thus from Eqs. 16 and 17

(20) $$F_{Abs}•F_{G}=F∕F_{H}$$

Bioavailability can be estimated either by determining dose corrected AUC_0→∞_ measures following oral and iv bolus dosing and/or dose corrected measures of unchanged drug in urine (U_∞_), by assuming that clearance is unchanged between the iv and oral dosing routes. There is a complication with the calculations above related to the assumption that clearance is the same between dosing routes, and this complication was not recognized prior to our discovery (2, 5) that pharmacokinetic derivations of clearance could be accomplished using in series and parallel rate-defining processes (Eq. 15). If following oral dosing, CL_absorption site_ is much greater than CL_system iv bolus_, then the procedures above are valid as clearance between iv and oral dosing routes are equivalent. However, if CL_absorption site_ is not significantly greater than CL_system iv bolus_, then from Eq. 15, CL_system oral dose_ will be smaller than CL_system iv bolus_. Therefore, calculations of bioavailability using systemic concentration measurements may be inaccurate in such cases. This can be conceptually explained for a slow in series oral input for which the measured systemic AUC will be increased compared to the same dose administered intravenously, analogous to the example discussed earlier, very slow metabolic clearance will rate limit subsequent metabolic steps.

In Wakuda *et al.* (7), we explained how slow input from the site of administration can result in systemic bioavailability values greater than unity, which are statistically significantly higher than bioavailability values derived from measurement of unchanged drug in the urine. These differences are subsequently reflected in statistically significant renal clearance values that are a function of route of dosing.

## Making Clinical Drug Dosing Decisions by Applying Clearance Equations from Rate-Defining Parallel and In Series Processes

We have now simply derived key pharmacokinetic equations using rate-defining parallel and in series processes adapted from Kirchhoff’s Laws, independent of differential equations or any mechanistic assumptions. We will apply these relationships to analyze the pharmacokinetics of an example hypothetical drug, KL25A, for which intravenous and oral data in humans are examined. The following analysis will focus on the key concepts in clinical pharmacokinetics that are essential for making drug dosing decisions, and how to adjust dosing due to changes in pharmacokinetics, such as those caused by drug-drug interactions, or changes in clearance due to disease states and other pharmacogenomic and physiologic variables.

## Analyzing Pharmacokinetic Data: iv Bolus Dosing

**Fig. 2:**
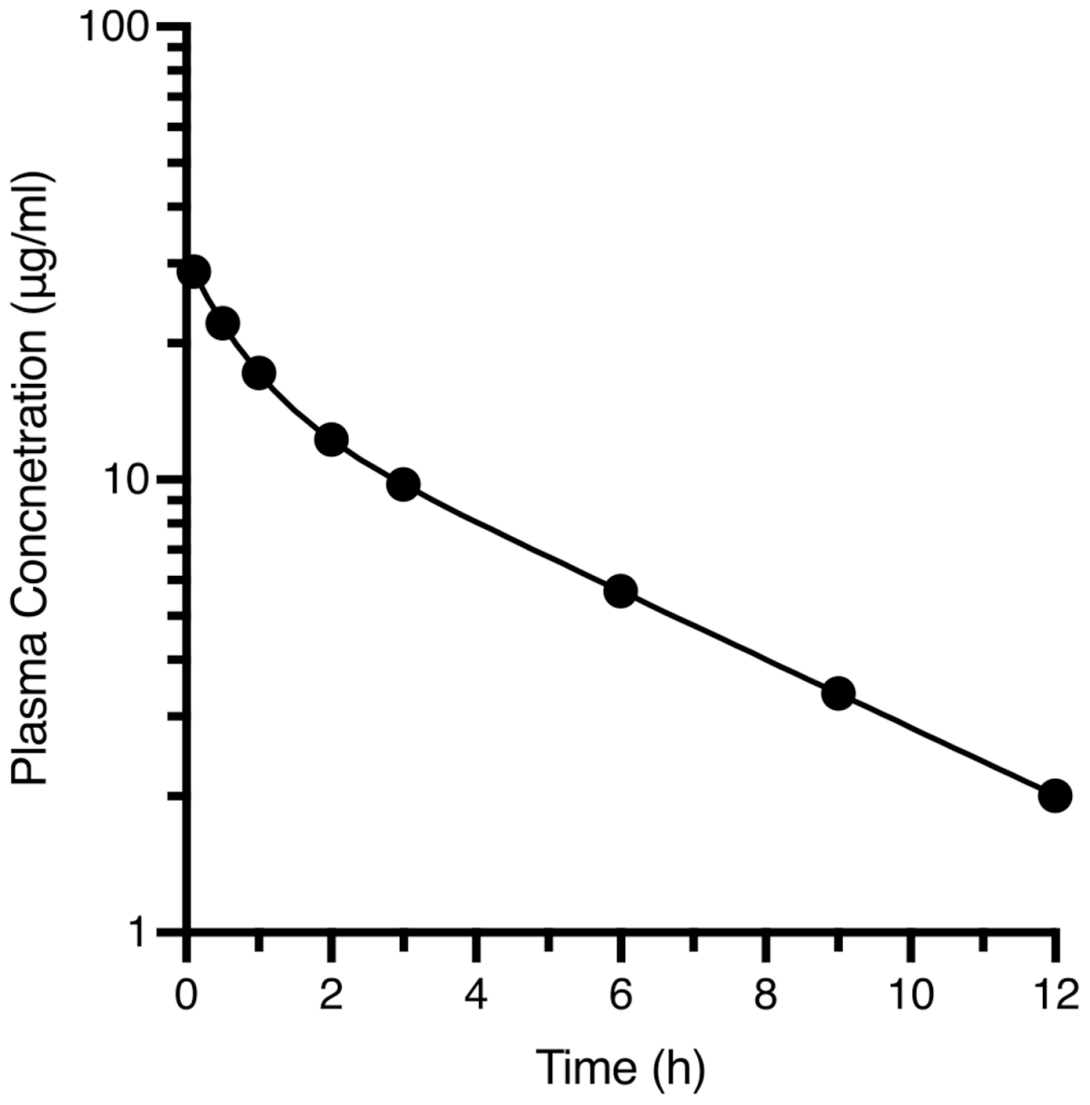
Semilogarithmic plasma concentration–time curve following an intravenous 2.5 gm bolus dose of KL25A to a 70 kg man. Data best computer fit to a biexponential equation $C_{p}=15•e^{-1.39t}+16•e^{-0.173t}$

Having completed derivation of the basic equations using parallel and in series rate defining processes, we begin analyzing pharmacokinetic data starting with iv bolus dosing of a hypothetical drug, KL25A, that follows linear kinetics. A typical logarithmic concentration time curve is depicted in Fig. 2 for a 250 mg iv bolus dose in a healthy volunteer.

## What is the Explanation for the Curvature in the Log Concentration Time Plot?

**Fig. 3:**
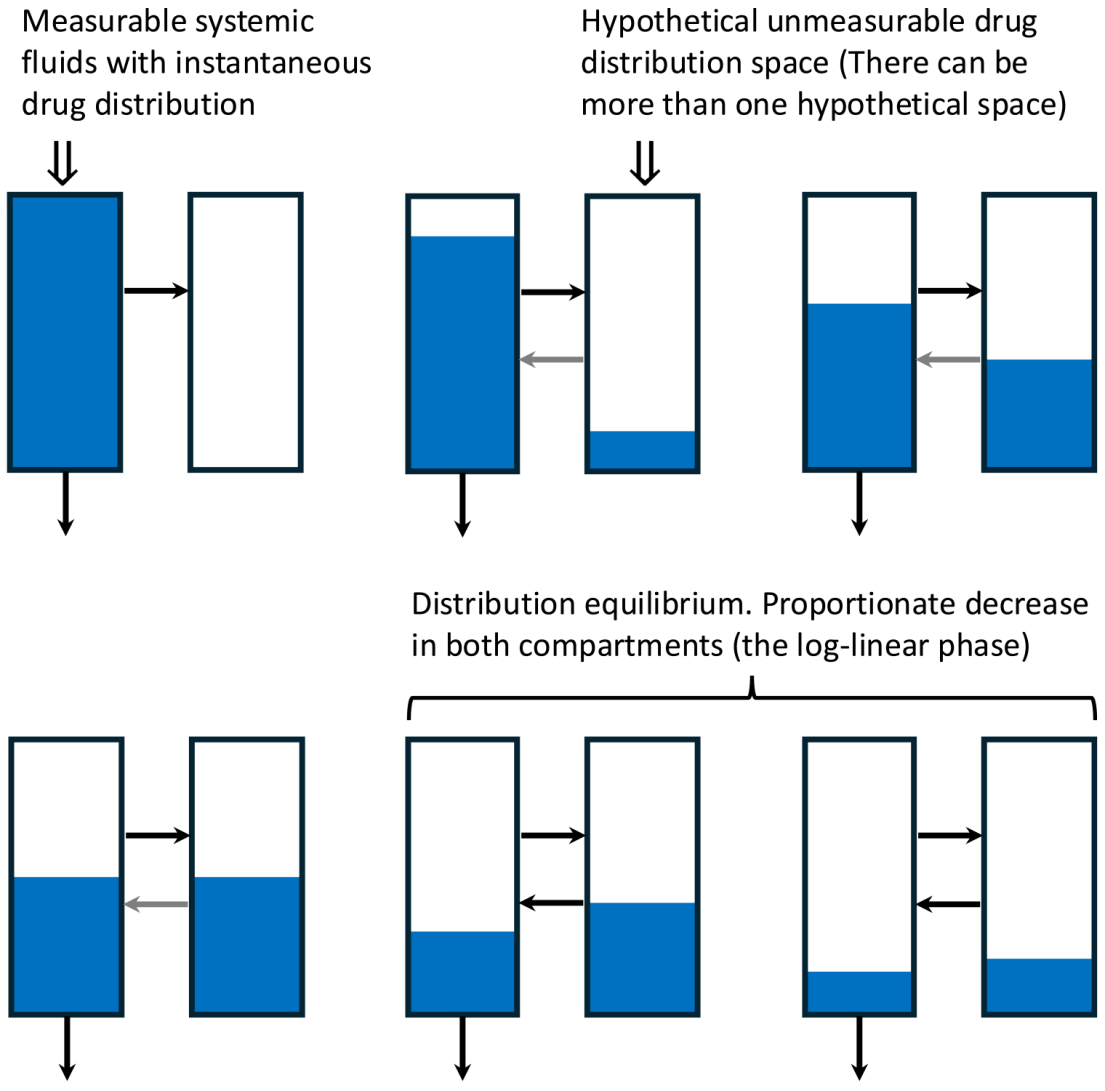
Representation of the time dependent dynamic changes of distribution following iv bolus administration of a drug that follows linear kinetics and exhibits a two or more exponential fit of the data

When an iv bolus dose is administered, cardiac output is so fast, 4.5 L/min, systemic concentrations will be uniform (assumed to be instantaneous), as depicted in the left hand rectangle in the upper far left of Fig. 3

Then as depicted in Fig. 3, concentrations in the systemic fluids will decrease as a function of two processes, drug elimination and drug distribution into unmeasureable lipophilic portions of the body that do not come into instantaneous distribution with the systemic circulation. This process of elimination and distribution out of the systemic circulation will continue, but as concentrations in the unmeasurable lipophilic portions of the body become greater, drug will also distribute back into the measurable systemic concentration. In Fig. 3, only one hypothetical unmeasurable compartment is depicted, but there can be more than one, dependent on the number of exponential terms that are required to define the drug concentration–time curve following iv bolus dosing. In the past, it has been taught that the number of exponentials required to fit the data is characterized in terms of multicompartment pharmacokinetics. However, the physiologic relevance to these hypothetical compartments is limited, and we suggest that the rationale for continuing to teach compartmental modelling may warrant reconsideration. In the last two bottom right depictions in Fig. 3, the ratio of concentrations in the hypothetical compartment to the measured systemic concentration become constant. This is representative of the log-linear phase of the concentration–time curve of Fig. 2, yielding the terminal exponential rate constant. Traditionally, students are taught to analyze the data to determine the coefficients and exponents of the equation describing the concentration time-curve, then calculating the AUC using the trapezoidal rule through the last measured concentration–time point, and then extrapolating to infinity by adding the concentration at the last measured concentration divided by the terminal log linear rate constant. While there may be educational value in understanding traditional approaches to illustrate foundational concepts, we believe they have little practical relevance today. We see limited value in continuing to teach this method, and instead advocate for using computer-based methods to fit the data – an approach that will inevitably become increasingly favored, as it aligns with the broader shift towards model-informed strategies in drug development.

## Determining AUC

Once the concentration–time equation for a drug following linear pharmacokinetics is known, here $C_{p}=15•e^{-1.39t}+16•e^{-0.173t}$, the value for AUC can be calculated (6) from the sum of the coefficients (L) divided by the exponents (λ) per Eq. 21, where n is the number of exponential terms giving the best fit of the data.


(21) $$AUC_{0\to \infty }=\Sigma_{i=1}^{n}\frac{L_{i}}{\lambda_{i}}$$

Thus, in the current example $AUC_{0\to \infty }=\frac{15}{1.39}+\frac{16}{0.173}=103\frac{\mu g•h}{ml}$

## Characterize the Elimination Parameters for iv bolus KL25A

The data obtained following the iv bolus dosing of KL25A as depicted in Fig. 2 are summarized in Table I.

### Determine the CL_P,total_, CL_P,R_ and CL_P,H_

Determine the plasma (P) clearance parameters. From Eq. 1 where for an iv dose F=1

$$CL_{P,total}=\frac{F•Dose}{AUC_{single\;dose\;0\to \infty }}=\frac{2,500\;mg}{(\frac{15}{1.39}+\frac{16}{0.173})\frac{mg•h}{L}}=24.2\frac{L}{hr}\;CL_{P,R}=\frac{U_{\infty }}{AUC_{single\;dose\;0\to \infty }}=\frac{1,430\;mg}{(\frac{15}{1.39}+\frac{16}{0.173})\frac{mg•h}{L}}=13.9\frac{L}{hr}\;CL_{P,H}=CL_{P,total}-CL_{P,R}=24.2-13.9=10.3\frac{L}{h}$$

However, to relate clearance measures to organ blood flow, plasma clearance values must be converted to blood clearance values using Eq. 22 and the blood to plasma concentration ratio, B/P, which for KL25A is 0.9 as given in Table I

(22) $${\mathrm{CL}}_{B}=\frac{{\mathrm{CL}}_{P}}{B∕P}$$

### Determine Elimination Characteristics of the Kidney

$CL_{B,R}=\frac{13.9}{0.9}=15.4\frac{L}{h}$ From Eq. 5 assuming kidney blood flow of 70 L/h.


$$\frac{1}{CL_{R}}=\frac{1}{Q_{R}}+\frac{1}{f_{uB}•GFR+(CL_{sec}-CL_{reab})};\frac{1}{15.4}=\frac{1}{70}+\frac{1}{0.1•7.2+(CL_{sec}-CL_{reab})}$$

Thus,

$$\left(CL_{sec}-CL_{reab}\right)=19.0\frac{L}{h}\;\text{and}\;CL_{filtration}=0.72\frac{L}{h}$$

Secretory clearance is the major renal route of elimination.

### Determine Elimination Characteristics of the Liver

$CL_{B,H}=\frac{10.3}{0.9}=11.4\frac{L}{h}$ It is unlikely that KL25A is a substrate for acid transporting hepatobiliary membrane OATPs with a B/P of 0.9. Acids generally do not distribute into blood cells and typically exhibit a B/P ≈ 0.55.

From Eq. 7, assuming liver blood flow of 90 $L∕\text{hr}\frac{1}{CL_{H}}=\frac{1}{Q_{H}}+\frac{1}{f_{uB}•CL_{H,int,(met+bil)}};\frac{1}{11.4}=\frac{1}{90}+\frac{1}{0.1•CL_{H,int,(met+bil)}}$

Thus, $CL_{H,int,(met+bil)}=13.1\frac{L}{h}$, and the primary route of hepatic elimination is biliary excretion, since only minor metabolites are measured.

Overall, renal clearance by transporters (19.0 L/h) is about 50% greater that biliary clearance (13.1 L/h), yet both the kidney and liver are relevant for KL25A elimination.

### Determine the Mean Residence Time of the Drug

For a drug following linear kinetics the mean residence time is calculated via Eq. 24, where AUMC (area under the moment curve, i.e., area under the product of C·t *vs* t) is the sum of the coefficients divided by the exponents squared (6) (Eq. 23) while Eq. 21 gives AUC.

(23) $$AUMC_{0\to \infty }=\sum_{i=1}^{n}\frac{L_{i}}{\lambda_{i}^{2}}$$

(24) $$\text{MRT}=\frac{AUMC_{0\to \infty }}{AUC_{0\to \infty }}=\frac{\frac{L_{1}}{\lambda_{1}^{2}}+\frac{L_{2}}{\lambda_{2}^{2}}}{\frac{L_{1}}{\lambda_{1}}+\frac{L_{2}}{\lambda_{2}}}=\frac{\frac{15}{1.39^{2}}+\frac{16}{0.173^{2}}}{\frac{15}{1.39}+\frac{16}{0.173}}=\frac{542}{103}=5.26\;h$$

*versus* the terminal elimination half-life of 0.693/0.173 = 4.01 h

### Determine the Volume of Distribution of the Drug

A single volume of distribution term is relevant (Eq. 25), designated previously as the volume of distribution steady-state (V_ss_), a volume term independent of elimination characteristic. It is an unfortunate accident that this volume term is designated as the volume of distribution steady-state, since it was first determined under steady-state conditions. In fact, V_ss_ has no relation with steady-state. It is the total volume in Fig. 3 for the measurable and unmeasurable compartments and represents this volume under all conditions. V_ss_ is the only relevant volume in clinical pharmacokinetics and it reflects the space in which the drug may distribute following an iv bolus dose.


(25) $$V_{ss}=CL•MRT=\frac{Dose•AUMC_{0\to \infty }}{{AUC_{0\to \infty }}^{2}}=\frac{24.2}{0.9}•5.26=142\;L$$

Multiplying measured systemic concentrations by this value gives the total amount of drug in the body. Traditional teaching of pharmacokinetics often includes extensive discussion of various volume parameters and the potential for protein binding within unmeasureable hypothetical compartments. However, we find limited clinical relevance in these analyses and instead view V_ss_ as simply providing an important measure of the theoretical space available for drug distribution, while also allowing for the examination of changes in this measure as a result of drug-drug interactions. It is recognized that when drug-drug interactions only involve changes in measures of metabolic elimination, V_ss_ is unchanged (8). However, when drug interactions involve transporters, then changes in V_ss_ and CL can both occur (9). For example, OATP1B inhibition by single-dose rifampin significantly decreased both clearance and volume of distribution for fluvastatin, rosuvastatin, atorvastatin, and glyburide (9, 10). In some cases, the decrease in volume was greater than the decrease in clearance, leading to the paradoxical result that decreased clearance is accompanied by a shorter half-life (10).

## Analyzing Pharmacokinetic Data: Oral Dosing

**Fig. 4:**
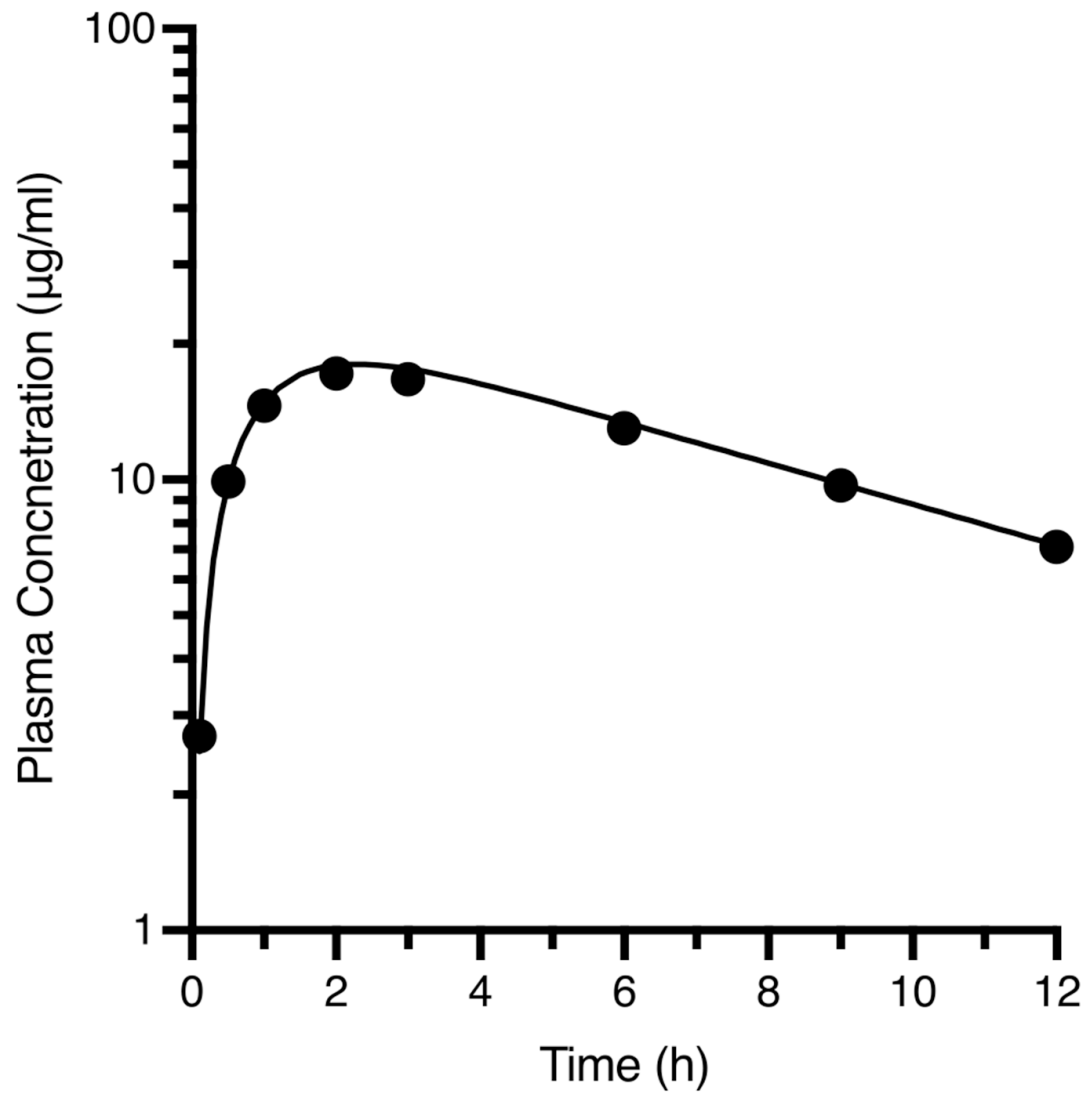
Semilogarithmic plasma concentration–time curve following a 5.0 gm oral dose of KL25A to a 70 kg man. Data best computer fit to a biexponential equation $C_{p}=22.7•e^{-0.105t}-22.7•e^{-1.16t}$

A 5 g oral dose of KL25A was given to the same subject with the plasma concentration time curve depicted in Fig. 4. The oral plasma concentration–time data were also best described by a biexponential equation, $C_{p}=22.7•e^{-0.105t}-22.7•e^{-1.16t}$.

## Characterize the Elimination Parameters for Oral KL25A

The data obtained following the oral dosing of KL25A as depicted in Fig. 4 are summarized in Table I.

### a. Determine bioavailability using both unchanged drug in urine and systemic concentrations correcting for dose differences


$$\;F_{urine\;data}=\frac{U_{\infty ,po}}{U_{\infty ,iv}}•\frac{Dose_{iv}}{Dose_{po}}=\frac{220}{1,430}•\frac{2,500}{5,000}=0.077\;F_{plasma\;data}=\frac{AUC_{0\to \infty ,po}}{AUC_{0\to \infty ,iv}}•\frac{Dose_{iv}}{Dose_{po}}=\frac{\frac{22.7}{0.105}-\frac{22.7}{1.16}}{103}•\frac{2,500}{5,000}=0.956$$

This marked difference in F is characteristic of the impact that slow drug delivery clearance has on influencing systemic AUC measurements, while having no effect on the actual amount of drug reaching the systemic circulation.

### b. Determine MAT for the Oral Dose

From Eq. 11 $MAT=MRT_{oral\;dose}-MRT_{iv\;bolus}=\frac{\frac{22.7}{0.105^{2}}-\frac{22.7}{1.16^{2}}}{\frac{22.7}{0.105}-\frac{22.7}{1.16}}\;5.26=\frac{2,042}{196.6}-5.26=5.13\;h$

Note that MAT for the oral dose is almost the equivalent for total MRT of the iv dose. The first order absorption rate constant determined as the inverse of MAT is 0.195 h^−1^, equivalent to an absorption half-life of 3.63 h.

## Lessons from the Pharmacokinetic Analysis and Why the KL25A Demonstration was Chosen

Having discovered that slow drug input into the systemic circulation can affect measured AUC, based on derivation of pharmacokinetic equations using parallel and in series rate-defining processes adapted from Kirchhoff’s Laws, we presented a hypothetical drug with an intermediate clearance (both by renal and hepatic processes) of 26.9 L/h following iv dosing. However, because of its polarity, KL25A was very poorly absorbed (F_urine data_ = 7.7%), and because absorption was so slow, the increase in AUC made it appear that oral bioavailability was almost complete (F_plasma data_ = 95.6%). Since pharmacodynamics is almost always a function of systemic exposure, the magnitude of pharmacodynamic outcome of this oral dose would be expected to be indistinguishable from that of the iv dose, based on the comparable systemic exposures between dosing routes, even in cases of indirect pharmacological mechanisms where the pharmacodynamic time course does not mirror the pharmacokinetic profile. We now translate these findings to an example of immediate release (IR) *vs* extended release (ER) oral dosage forms (from a publication in preparation).

As depicted in Table II, Lukacsko *et al.* (11) report a significant decrease in LDL cholesterol and total cholesterol for 149 hypercholesterolemic patients receiving 20 mg daily doses of ER lovastatin *vs* 20 mg daily IR lovastatin. Systemic lovastatin concentrations were not measured in this study. However, 2 years earlier the company reported (12) that daily 20 mg doses of ER lovastatin *vs* IR lovastatin, in a different population, gave a 71% average increase in lovastatin AUC at day 28 as given in Table III.

These data are consistent with the KL25A demonstration above, where slow drug clearance from the absorption site leads to a marked increase in AUC as compared to a comparable iv bolus dose. These findings may lead to a paradigm shift in drug development of NMEs as we recently proposed (13). Drug developers have often considered molecules with low clearance and longer half-life advantageous, particularly for increasing the likelihood of achieving projected efficacious concentrations and desired pharmacodynamic effects in humans. But then major Pharma is ignoring its greatest strengths, drug development and expertise in formulation. The KL25A and lovastatin examples presented here suggests that moderate or high clearance drugs may be preferable because then systemic clearance can be controlled by the formulation, essentially independent of mechanisms of drug elimination and patient elimination process variability. We used the hypothetical KL25A in the demonstration since then it was possible to quantitate the advantage with plasma *versus* urine measurements. However, the process holds also for highly metabolized drugs administered by non-oral routes (e.g., SubQ).

## Clinical Pharmacokinetic Equations Derived by Adapted Kirchhoff’s Laws

In this tutorial we have shown that all relevant hepatic and renal clearance equations may be simply derived independent of differential equations based on understanding parallel and in series rate-defining processes that include all potential drivers including organ blood flow and drug delivery kinetics. There are no mechanistic implications related to the organs of elimination, consistent with the fact that clinically only systemic concentrations and urine amounts can be measured. As we have previously shown (3-5, 14, 15), when hepatobasolateral membrane transporters are not clinically relevant following iv bolus dosing Eq. 7 leads to

(26) $$CL_{H}=Q_{H}•\frac{f_{uB}•CL_{int}}{Q_{H}+f_{uB}•CL_{int}}$$

which for the past 50 years had been incorrectly characterized as the well-stirred model (WSM) of hepatic elimination but was derived (2-5) independent of any mechanistic model of hepatic elimination. This explains why all isolated perfused liver studies are only consistent with Eq. 26 (16, 17). Furthermore, when basolateral transport is clinically relevant and hepatic blood flow is markedly greater than $f_{\text{uB}}\cdot {\text{CL}}_{\text{int}}$ and $f_{\text{uB}}\cdot ({\text{CL}}_{H,\text{int},\text{influx}}-{\text{CL}}_{H,\text{int},\text{efflux}})$, following iv bolus dosing Eq. 7 leads to

(27) $${\mathrm{CL}}_{H}=\frac{f_{\mathrm{uB}}•{\mathrm{CL}}_{\mathrm{int}}•({\mathrm{CL}}_{H,\mathrm{int},\mathrm{influx}}-{\mathrm{CL}}_{H,\mathrm{int},\mathrm{efflux}})}{{\mathrm{CL}}_{\mathrm{int}}+({\mathrm{CL}}_{H,\mathrm{int},\mathrm{influx}}-{\mathrm{CL}}_{H,\mathrm{int},\mathrm{efflux}})}=\frac{f_{\mathrm{uB}}•({\mathrm{CL}}_{H,\mathrm{int},\mathrm{influx}}-{\mathrm{CL}}_{H,\mathrm{int},\mathrm{efflux}})}{1+\frac{\left({\mathrm{CL}}_{H,\mathrm{int},\mathrm{influx}}-{\mathrm{CL}}_{H,\mathrm{int},\mathrm{efflux}}\right)}{{\mathrm{CL}}_{\mathrm{int}}}}$$

which is the correct equation to evaluate the effect of hepatobiliary transporters rather than the extended clearance model (5, 18). When drug input is not iv bolus dosing, renal and hepatic clearance equations must also include the clearance from the delivery site in the CL_entering_ portion.

## Renal and Hepatic Clearance Equations

### Linear Systems

Thus, the equations for renal and hepatic clearance for linear systems are given, respectively, in Eqs. 28 and 29 as

(28) $$\frac{1}{CL_{R}}=\frac{1}{CL_{deliverysite}}+\frac{1}{Q_{R}}+\frac{1}{f_{uB}•GFR+(CL_{secretion}-CL_{reabsorption})}$$

(29) $$\frac{1}{CL_{H}}=\frac{1}{CL_{deliverysite}}+\frac{1}{Q_{H}}+\frac{1}{f_{uB}•(CL_{H,int,influx}-CL_{H,int,efflux})}+\frac{1}{f_{uB}•CL_{int}}$$

When an iv bolus dose of drug is administered, input is instantaneous and therefore $CL_{deliverysite}$ is infinite, that inverse term disappearing from Eqs. 28 and 29.

### Nonlinear Saturable Processes

As we have shown in our ISSX abstract (19) and Pharmacological Reviews paper (5), with more detail provided in a manuscript under review, for any nonlinear saturable elimination or absorption process, the relevant clearance parameter is characterized as

(30) $$\frac{1}{CL_{saturable}}=\frac{1}{\frac{V_{max}}{\left[S_{50}\right]}}+\frac{1}{\frac{V_{max}}{\left[S\right]}}$$

In Eq. 30, the two rate-defining processes are a) the linear clearance observed at low substrate concentrations, $\frac{V_{max}}{\left[S_{50}\right]}$, when clearance is first order and Rate of elimination = $\frac{V_{max}}{\left[S_{50}\right]}•[S]$ and b) the zero order clearance observed at very high substrate concentrations, $\frac{V_{max}}{\left[S\right]}$, and Rate of elimination = $V_{max}$, where V_max_, the maximum rate of drug elimination, has units of mass/time; [S_50_] is the substrate concentration at half V_max_, a constant with units of mass/volume; and [S] is the variable substrate concentration.

## Making Drug Dosage Decisions in Clinical Medicine

Depending on the systemic concentration believe to be clinically relevant for effective and safe therapeutics, doses and timing of dosing are adjusted to equal the rate of elimination determined by the relevant clearance equation(s) above (Eqs. 26-29) multiplied by that desired concentration.

## Conclusions

All relevant clearance equations can be simply derived independent of differential equations, based on understanding parallel and in series rate-defining linear and nonlinear processes that include all potential drivers, including organ blood flow and drug delivery kinetics. There are no mechanistic implications related to the specific organs of elimination, which is consistent with the clinical reality that only systemic concentrations and urine amounts can be measured. Although some leading scientists remain unconvinced of the advantages of our approach to deriving clearance equations independent of differential equations, we are unaware of any evidence demonstrating that clinical data are incorrectly analyzed by the approach proposed here. In contrast, there are considerable clinical data, as detailed above, that can only be explained by the approach proposed here.

## Figures and Tables

**Table I:** Pharmacokinetic Information Describing iv Bolus (Fig. 2) and Oral (Fig. 4) Dosing of KL25A to a Healthy Subject

Parameter (Unit)	iv Bolus	Oral
Dose (gm)	2.5	5
Plasma Concentration (μg/ml)-Time (h) Equation	$C_{p}=15•e^{-1.39t}+16•e^{-0.173t}$	$C_{p}=22.7•e^{-0.105t}-22.7•e^{-1.16t}$
U_∞_: Parent Drug Excreted Unchanged (gm)	1.43	0.220
U_∞,met_: Metabolites Excreted in Urine (gm)	0.024	0.004
f_uB_	0.10	0.10
GFR: Glomerular Filtration Rate (ml/min)	120	120
B/P: Blood-to-Plasma Concentration Ratio	0.9	0.9

**Table II:** Inferential Analysis Results for Percent Change in LDL, HDL, Total Cholesterol, and Triglycerides from Baseline to Endpoint in 20 mg Population, Adapted from Lukacsko *et al.* (11)

Treatment 20 mg (*n* = 149)	Percent Change LS Mean ± SE (95%) CI			
	LDL Cholesterol	HDL Cholesterol	Total Cholesterol	Triglycerides
ER Lovastatin	−26.4 ± 1.06 (–28.5, –24.3)	4.1 ± 1.04 (2.0, 6.1)	**−19.1 ± 0.83** **(−20.7, −17.4)**	**−7.4 ± 2.14** **(−11.7, −3.2)**
IR lovastatin	−23.1 ± 1.06 (–25.2, –21.0)	4.3 ± 1.04 (2.3, 6.4)	−17.2 ± 0.83 (−18.8, −15.5)	−10.4 ± 2.14 (−14.7, −6.2)
ER *vs* IR	−3.3 ± 1.08	−0.2 ± 1.28	−1.9 ± 0.91	3.0 ± 2.81
	p = 0.0028	p = 0.8584	p = 0.0355	p = 0.2867

All numbers are from a two-way ANOVA for percent change with treatment and center as factors CI, confidence interval; ER, extended release; IR, immediate release; LS, least squares; SE, standard error

**Table III:** Area Under the Plasma Concentration–Time Curve from Zero to 24 Hours (AUC_0–24_) for Lovastatin, Lovastatin Acid, and Total and Active Inhibitors of 3-Hydroxy-3-Methylglutaryl Coenzyme A (HMG-CoA) Reductase with 20 mg Extended Release and 20 mg Immediate Release Lovastatin, Adapted from Davidson *et al.* (12)

AUC_0–24_ (ng • h/mL) (mean ± SD)	Lovastatin ER			Lovastatin IR		
	Day 1	Day 28	R	Day 1	Day 28	R
Lovastatin	49.9 ± 23.5	76.6 ± 36.9	1.48	33.7 ± 21.6	44.7 ± 46.2	1.15
Lovastatin acid	38.6 ± 31.4	87.1 ± 67.2	1.88	84.1 ± 63.1	82.5 ± 60.3	1.00
Total inhibitors of HMG-CoA reductase	136.3 ± 73.3	262.6 ± 159.4	1.81	226.9 ± 100.4	251.6 ± 154.1	1.06
Active inhibitors of HMG-CoA reductase	83.3 ± 44.7	171.3 ± 122.9	1.86	178.9 ± 82.9	185.9 ± 100.4	1.02

R = accumulation ratio (geometric mean ratio of C_max_ on day 28 to C_max_ on day 1)

ER, extended release; IR, immediate release; SD, standard deviation

## Data Availability

All data generated or analyzed as part of this tutorial are included in the article or in the references provided.

## Abbreviations

AUC: Area under the systemic concentration–time curve
AUMC: Area under the moment curve
BCRP: Breast cancer resistance protein
B/P: Blood to plasma concentration ratio
C_B_: Drug concentration in blood
C_P_: Drug concentration in plasma
CL: Clearance
CL_absorption site_: Clearance of drug from the absorption site
CL_bil_: Clearance of drug by biliary excretion
CL_deliverysite_: Clearance of drug from the delivery site
CL_entering_: Entering clearance
CL_filtration_: Renal filtration clearance
CL_H_: Hepatic clearance
CL_H,efflux_: Hepatobiliary membrane efflux clearance
CL_H,influx_: Hepatobiliary membrane influx clearance
CL_int_: Intrinsic clearance
CL_leaving_: Leaving clearance
CL_met_: Clearance of drug by hepatic metabolism
CL_R_: Renal clearance
CL_R,reabsorption_: Renal tubular reabsorption clearance
CL_R,secretion_: Renal tubular secretion clearance
CL_saturable_: Nonlinear saturable drug clearance
CL_system iv bolus_: Systemic clearance measured following an iv bolus dose
CL_system oral bolus_: Systemic clearance measured following an oral dose
CYP3A: Cytochrome polypeptide 3A
ER: Extended release
ExR_H_: Hepatic extraction ratio
F_Abs_: Fractional bioavailability due to absorption
F_G_: First pass fractional gastrointestinal bioavailability
F_H_: First pass fractional hepatic bioavailability
F_plasma data_: Bioavailability calculated using systemic plasma concentration measurements
f_uB_: Fraction unbound in the blood
F_urine data_: Bioavailability calculated based on amounts of unchanged drug in the urine
GFR: Glomerular filtration rate
IM: Intramuscular
IR: Immediate release
IVIVE: *In vitro-in vivo* extrapolation
k_absorption_: First-order absorption rate constant
k_system iv bolus_: Single rate constant representing systemic elimination following an iv bolus dose
k_system oral bolus_: Single rate constant representing systemic elimination following an oral dose
L: Coefficient in equation defining best fit of the systemic concentration–time curve
LDL: Low density lipoprotein
MAT: Mean absorption time
MRT: Mean residence time
n: Number of exponential terms in equation defining best fit of the systemic concentration–time curve
NME: New molecular entity
OATP: Organic anion transporting polypeptide
P-gp: P-glycoprotein
Q_H_: Hepatic blood flow
Q_R_: Renal blood flow
SubQ: Subcutaneous
t: Time
U_∞_: Total amount of unchanged drug excreted in the urine
U_∞,met_: Total amount of drug metabolites excreted in the urine
UGTs: Uridine glucuronosyltranferases
V_absorption site_: Volume of distribution of drug in the absorption site
V_max_: Maximum rate of drug elimination when metabolism is saturated
V_ss_: Volume of distribution steady state
WSM: Well stirred model
λ: Exponent in equation defining best fit of the systemic concentration–time curve
[S]: Substrate concentration
[S_50_]: Substrate concentration at half V_max_
